# Advancing 1.5T MR imaging: toward achieving 3T quality through deep learning super-resolution techniques

**DOI:** 10.3389/fnhum.2025.1532395

**Published:** 2025-06-18

**Authors:** Sk Rahatul Jannat, Kirsten Lynch, Maryam Fotouhi, Steve Cen, Jeiran Choupan, Nasim Sheikh-Bahaei, Gaurav Pandey, Bino A. Varghese

**Affiliations:** ^1^Department of Radiology, University of Southern California, Los Angeles, CA, United States; ^2^Department of Neurology, University of Southern California, Los Angeles, CA, United States; ^3^Department of Genetics & Genomic Sciences, Icahn School of Medicine at Mount Sinai, New York, NY, United States

**Keywords:** image quality, super resolution, T1 weighted, Image harmonization, transformer enhanced GAN

## Abstract

**Introduction:**

A 3T MRI scanner delivers enhanced image quality and SNR, minimizing artifacts to provide superior high-resolution brain images compared to a 1.5T MRI. Thus, making it vitally important for diagnosing complex neurological conditions. However, its higher cost of acquisition and operation, increased sensitivity to image distortions, greater noise levels, and limited accessibility in many healthcare settings present notable challenges. These factors impact heterogeneity in MRI neuroimaging data on account of the uneven distribution of 1.5T and 3T MRI scanners across healthcare institutions.

**Methods:**

In our study, we investigated the efficacy of three deep learning-based super-resolution techniques to enhance 1.5T MRI images, aiming to achieve quality analogous to 3T scans. These synthetic and “upgraded” 1.5T images were compared and assessed against their 3T counterparts using a range of image quality assessment metrics. Specifically, we employed metrics such as the Structural Similarity Index Measure (SSIM), Peak Signal-to-Noise Ratio (PSNR), Learned Perceptual Image Patch Similarity (LPIPS), and Intensity Differences in Pixels (IDP) to evaluate the similitude and visual quality of the enhanced images.

**Results:**

According to our experimental results it has been exhibited that among the three evaluated deep learning-based super-resolution techniques, the Transformer Enhanced Generative Adversarial Network (TCGAN) significantly outperformed the others. To reduce pixel differences, enhance image sharpness, and preserve essential anatomical details TCGAN performed efficaciously.

**Discussion:**

This approach presents TCGAN offers a cost-effective and widely accessible alternative for generating high-quality images without the need for expensive, high-field MRI scans and leads to inconsistencies and complicate data comparison and harmonization challenges across studies utilizing various scanners.

## 1 Introduction

Magnetic resonance imaging (MRI) is widely used in neuroimaging due to its non-invasive nature, excellent soft tissue contrast, and painless procedure. While 1.5T MRI is the current clinical standard, 3T MRI is gaining popularity for providing clearer images, increased spatial resolution, decreased scan time, higher detection of pathologies particularly small pathologies, better delineation of small structures of the brain and better gray-white matter contrast, which aids brain tissue segmentation, better delineation of small structures of the brain etc. Despite the growing availability of 3T MRI, most clinical scanners in the U.S. remain 1.5T.^1^^,^^2^ Though 3T MRI offers higher resolution, it cannot be used in certain cases, such as patients with metallic implants, those with heat sensitivity, and pregnant women. It is also less suitable for areas prone to artifacts and certain imaging like cardiac or abdominal scans (Graves, 2022). Upgrading 1.5T images to 3T quality could significantly improve diagnosis and treatment decisions but replacing all 1.5T systems with 3T is expensive and complex. This scenario leads to unresolved heterogeneity in MRI neuroimaging data from the disparate distribution of 1.5T and 3T MRI scanners across healthcare settings, which in turn creates major hurdles when pooling data from different MRI centers. For example, challenges due to differences in field strength can increase the disparity in detecting small structures and neurological pathologies. More importantly in longitudinal follow ups of cases and comparison with prior studies to detect changes over the time. This difference in imaging quality can create inconsistencies, complicating data comparison and integration from studies utilizing varying scanner types, particularly in longitudinal imaging efforts, such as the Alzheimer’s Disease Neuroimaging Initiative (ADNI) (Pomponio et al., 2020). Further complicating matters, the lack of standardization in image acquisition protocols and post-processing techniques raises questions about the reliability of conclusions drawn from studies using mixed field strengths.

To leverage the strengths of both 1.5T and 3T MRI, the generation of super-resolution images from 1.5T scans that approach the quality of 3T images is essential. Recent advancements in deep learning, particularly convolutional neural networks (CNNs), have enabled high-resolution image generation from lower-resolution MRI scans, harmonizing studies with mixed field strengths. While Pham et al. showed that Super-Resolution Convolutional Neural Network (SRCNN) (Pham et al., 2017) effectively enhances low-resolution brain images using high-resolution patches, Chen et al. (2018) introduced a GAN-guided 3D network that produced high-quality images six times faster for degraded images. Studies by Soltanmohammadi and Faroughi (2023) indicated that Super-Resolution GAN (SRGAN) and Efficient Sub-pixel CNN (ESPCN) outperform SRCNN in generating non-medical high-resolution images. On similar lines, Liao et al. (2022) demonstrated that SRGAN, ESPCN, and UNet architectures excel in enhancing brain MR images acquired from ADNI 1 dataset, which includes 47 subjects. More recently, Li et al. (2022) used a Transformer-Enhanced GAN (TCGAN) integrated with Transformer architecture in generator to generate synthetic images of T1- (T1w) and T2-weighted (T2w) images and reported on its superior performance compared to other CNN and GAN methods. Interpolation methods, including bicubic and Lanczos interpolation, remain effective alternatives for resolution enhancement, as shown by Shelomentseva (2023) and Jahnavi et al. (2024). However, in contrast to deep learning methods, while interpolation methods offer quick and less computationally demanding solutions, the resultant images suffer from blurred edges and a lack of detail. Also, while traditional methods are readily available and easily applied to any image, deep learning techniques excel in critical clinical applications by handling noise and complexity in low-field MRI scans. Currently, there is no consensus regarding the optimal method for super-resolution tasks, the choice between these methods depends on the specific context, balancing the need for image quality against resource availability and technical capabilities. Although deep learning models can generate high-resolution images, it is essential to quantify these improvements and assess their applicability for clinical goals. For instance, the impact of mixed MRI field strengths on radiomics (Savadjiev et al., 2019) is complex, affecting feature extraction, data integration, and clinical applicability. Hodneland et al. found that a significant proportion of extracted radiomic features were statistically associated with specific MRI protocols, with features being influenced by up to 62% due to differences in field strength for certain cancers (Hodneland et al., 2024). Additionally, Ammari et al. demonstrated that 10 out of 15 analyzed radiomic features showed significant differences between 1.5T and 3T MRI scans (Ammari et al., 2021). Addressing these challenges through standardization and normalization methods is crucial for ensuring that radiomics can fully realize its potential as a powerful tool in diagnostic and prognostic applications across diverse imaging environments.

Several image quality assessment metrics are employed in MRI applications; however, they come with limitations, particularly in the context of radiomics (Kastryulin et al., 2023). Full-reference image quality metrics are critical tools in evaluating the quality of images by comparing them to a known distortion-free reference image. They are widely used in various imaging applications, including MRI, as they provide quantitative assessments of how closely a degraded image resembles an ideal version. Some of the commonly used full-reference image quality metrics include well-established metrics such as Structural Similarity Index Measure (SSIM), Peak Signal-to-Noise Ratio (PSNR) and Learned Perceptual Image Patch Similarity (LPIPS). While full-reference metrics are fundamental for assessing image quality, the metrics generally focus on global features and may overlook local variations critical for medical images, where localized pathology may be significant such as radiomics. Additionally, there are more advanced image quality metrics such as universal image quality index (UIQ), visual information fidelity (VIF), visual signal-to-noise ratio (VSNR) etc. (Pedersen and Hardeberg, 2012), that also account for local variations that are warranted for use in radiomics applications, where the accuracy of feature extraction is vital for clinical decision-making.

To address some of the limitations in the current literature, in this study, we evaluated three deep learning based super-resolution (SR) models (TCGAN, SRGAN, ESPCN) and two interpolation methods (Bicubic and Lanczos) to generate synthetic 3T T1-weighted brain images from 1.5T T1-weighted scans using unique 3T-1.5T image pairs obtained from the same group of patients in the ADNI dataset. Our goal was to determine how well these models could enhance the image quality of 1.5T images to match typical 3T scanner outputs. Also, we employed well-established metrics such as Structural Similarity Index Measure (SSIM), Peak Signal-to-Noise Ratio (PSNR) and Learned Perceptual Image Patch Similarity (LPIPS) to measure the fidelity and visual quality of the generated 3T images. Additionally, we present a novel metric for image quality assessment, namely “Intensity Differences in Pixels (IDP)” to assess both the local and global variations and evaluate its performance in differentiating the performance of the 5 SR models.

## 2 Materials and methods

### 2.1 Image acquisition

In this study, we utilized data from the publicly available ADNI (IDA, 2025) database. We focused on a subset of 163 participants who had both 1.5T and 3T T1-weight MRI scans, allowing for a quantitative comparison between the synthetic 3T images generated from the 1.5T scans and the real 3T scans. Only subjects scanned using three major MRI manufacturers namely GE, SIEMENS, and Philips were considered (Table 1). Supplementary Table 1 provides all vendor-specific acquisition parameters. No cases with cross- manufacturer data were used for generating synthesized 1.5T images in comparison with 3T images. This decision was made due to signal-to-noise ratio (SNR) and contrast-to-noise ratio (CNR) differences between MRI field strengths, specifically between 1.5T and 3T, without addressing the broader variations in image reconstruction methods used by different MRI manufacturers (Kushol et al., 2023). Each manufacturer uses unique algorithms and parameters to reconstruct images, which can introduce variability in image appearance and quality beyond the differences due to field strength alone. To ensure consistency across datasets and to prevent confounding variables related to field strength and noise processing, we excluded cases where images were acquired at 1.5T from GE/Siemens and 3T from Philips, as well as the reverse scenario where 1.5T images came from Philips and 3T from GE/Siemens.

**TABLE 1:** Subject count by manufacturer.

Manufacturer name	#Number of subjects
Siemens-Siemens	99
GE –GE	32
Philips-Philips	32
Cross manufacturers	37

We trained the Super Resolution (SR) models using the ADNI 1 dataset, which contains 14,080 axial T1 images. Due to differences in the acquisition processes between 1.5T and 3T MRI scans, misalignment was common, with anatomical structures not perfectly overlapping. Such misalignment can introduce significant errors and inconsistencies when comparing or combining data from these two sources. To mitigate this issue and ensure accurate analysis, we realigned the 1.5T images to match the orientation of the 3T images. This alignment process was crucial for performing precise, slice-by-slice comparisons and for ensuring that the evaluation of the SR models was based on correctly corresponding anatomical regions in both image sets. The realignment was achieved using Statistical Parametric Mapping (SPM) 12 software (Penny et al., 2011).

**FIGURE 1:**
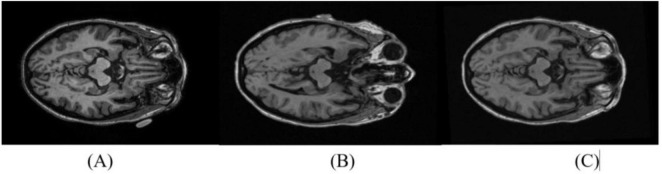
Reorientation of 1.5T images to match the 3T pair. A representative axial slice from the 3T scan is shown in **(A)**. The 1.5T slice pair is shown before **(B)** and after **(C)** reorientation.

In all cases, the image alignment using the SPM software resulted in 1.5T images with consistent anatomic correspondence to the 3T image pair. All 200 subject pairs were visually inspected to confirm accurate alignment. In cases where initial misalignment was observed, the realignment procedure was iteratively repeated for the affected pairs until the 1.5T images were correctly aligned with the 3T scans (Figure 1).

### 2.2 Super resolution models

The processed 1.5T T1-weight images were then used as inputs for three distinct super-resolution (SR) models and two interpolation methods, each designed to improve the spatial resolution of the medical images. By applying these advanced SR techniques, the goal is to capture finer anatomical details, potentially enhancing diagnostic accuracy and delivering more precise visual information for clinical assessments, all without the need for higher field strength re-scanning.

#### 2.2.1 Bicubic interpolation

Bicubic interpolation (Keys, 1981) resamples images by estimating new pixel values from a 4 × 4 grid of neighboring pixels, resulting in smoother and sharper visuals than simpler methods. It’s commonly used in fields like medical imaging due to its balance of quality and efficiency (Gavade and Sane, 2014). The synthesis formula for bicubic interpolation is as follows:


$$P\,\left(x,y\right)=\sum_{i=0}^{i=3}\sum_{j=0}^{j=3}a_{i\,j}\,x^{i}\,y^{j}$$

Here, P(x, y) = the interpolated values of (x, y) point and *a*_*ij*_ = coefficients determined by the cubic interpolation formula.

#### 2.2.2 Lanczos Interpolation

Lanczos interpolation (Duchon, 1979) Lanczos interpolation delivers sharp, high-quality images with minimal artifacts, ideal for precision tasks like scientific imaging. It resamples by evaluating pixels within a defined grid, balancing image quality and computational load for advanced image processing.

The interpolation formula for Lanczos is:


$$S\,\left(x\right)=\sum_{i=\left\lfloor x\right\rfloor -a+1}^{\left\lfloor x\right\rfloor +a}s_{i}\,L\,\left(x-i\right)$$

Where **”a”** represents the window size, **L (x - i)** is the Lanczos kernel, and **S(x)** is the interpolated value at position **x**. Lanczos is powerful for its ability to balance sharpness and smoothness, making it a preferred method for many advanced image processing applications (Turkowski, 1990).

#### 2.2.3 Efficient sub-pixel convolutional neural network

A specialized neural network architecture (Shi et al., 2016) uses a pixel-shuffling technique to convert low-resolution feature maps into high-resolution images efficiently. This sub-pixel convolution method minimizes computational costs while delivering high-quality results, making it ideal for applications in medical imaging, security, and computer vision (DDI, 2018).

**FIGURE 2:**
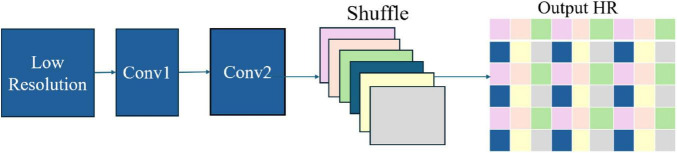
ESPCN architecture.

The ESPCN architecture (Figure 2) employs low- and high-resolution image pairs during training with a loss function, often based on Mean Squared Error, to enhance image reconstruction accuracy. This efficient design makes ESPCN well-suited for applications such as real-time video processing and medical imaging on resource-constrained devices.

#### 2.2.4 Super resolution generative adversarial network

A deep learning-based model (Ledig et al., 2017) that utilizes adversarial training to convert low-resolution images into high-resolution, realistic outputs. It comprises two main components: the Generator, which creates the upscaled images, and the Discriminator, which assesses their quality against real high-resolution images. This adversarial interplay enables the Generator to produce increasingly lifelike results through continuous feedback.

**FIGURE 3:**
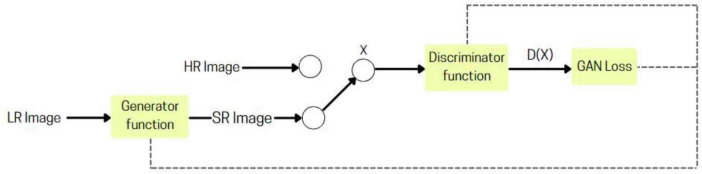
SRGAN architecture.

SRGAN’s (Figure 3), generator utilizes convolutional layers to extract details from low-resolution images, with batch normalization stabilizing training and residual blocks preserving key features. The Discriminator assesses authenticity through its convolutional layers and batch normalization, enhanced by Leaky ReLU activation functions. Adversarial learning during training drives the Generator to produce realistic high-resolution images, making SRGAN effective for video upscaling and medical imaging applications.

#### 2.2.5 TCGAN: transformer-enhanced GAN

TCGAN builds upon the principles of Generative Adversarial Networks (GANs) and comprises three key components: a transformer generator, a CNN-based generator, and a discriminator. The two generators are arranged in series to enhance performance and capabilities.

**FIGURE 4:**
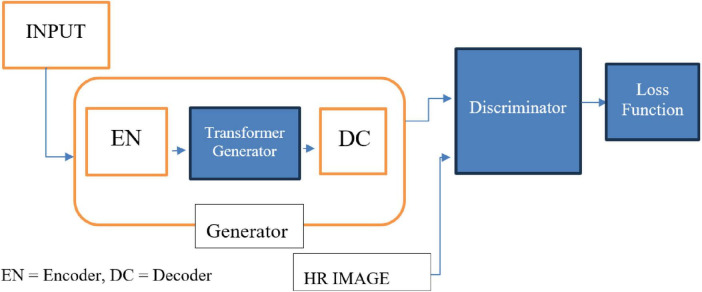
TCGAN architecture.

The TCGAN architecture (Figure 4) uses a hybrid generator combining transformer and CNN-based U-Net models (Vaswani, 2017), with attention mechanisms (Ronneberger et al., 2015) for capturing global information, enhancing synthesis quality. The Patch GAN discriminator evaluates image patches, promoting realistic, high-resolution image generation through adversarial training.

**FIGURE 5:**
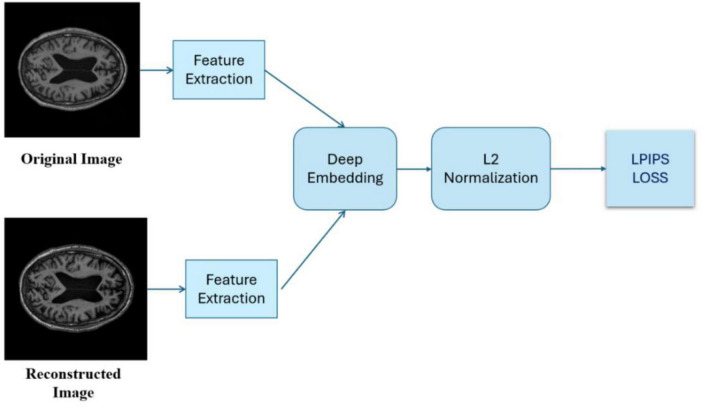
LPIPS loss is computed after extracting features and deeply embedding the images.

### 2.3 Assessment metrics

The synthesized 3T images generated by the above SR models were evaluated using several quantitative metrics.

#### 2.3.1 PSNR

PSNR is a key metric in image processing used to assess the quality of reconstructed or compressed images by comparing them to the original reference. It measures the ratio of maximum signal strength to noise introduced during compression, with higher values indicating better quality and less distortion. Expressed in decibels (dB), PSNR provides a quantitative measure to evaluate the performance of super-resolution models. The formula for PSNR is given as:


$$P\,S\,N\,R=20\,l\,o\,g\,10\,\left(M\,a\,x_{f}/\sqrt{M\,S\,E}\right)$$

Here *Max*_*f*_ represents the maximum possible pixel value of the original image. For example, in an 8-bit grayscale image, Max_f would be 255 (the highest pixel value). MSE is the mean squared error between the original image and the synthetic (or reconstructed) image. MSE is calculated by averaging the squared differences between corresponding pixels of the two images.

#### 2.3.2 Structural Similarity Index

The Structural Similarity Index (SSIM) (Wang et al., 2004) is more aligned with human perception, as it accounts for the human visual system’s sensitivity to structural features, brightness, and contrast. SSIM compares two images by evaluating three primary components:

• Luminance: The overall brightness or intensity of the pixels.
• Contrast: The difference between the darkest and brightest areas of the image.
• Structure: The spatial arrangement of pixel patterns, which represents the structural details in the image:

$$S\,S\,I\,M\,\left(x,y\right)=l\,{\left(x,y\right)}^{\alpha }.c\,{\left(x,y\right)}^{\beta }.s\,{\left(x,y\right)}^{\gamma }$$

Here, x and y are the two images being compared. l(x, y) measures luminance similarity between the two images. c(x, y) measures contrast similarity. s(x, y) measures structural similarity. α, β, and γ are weighting factors that control the importance of each component. A higher SSIM indicates greater similarity between the synthetic and original images.

#### 2.3.3 Learned perceptual image patch similarity

An advanced metric (Zhang et al., 2018) measures perceptual similarity between images by analyzing visual patterns and textures, rather than relying on pixel-based comparisons like PSNR and SSIM. Leveraging deep neural networks trained on image recognition, LPIPS assesses images based on how they appear to the human eye, capturing subtle perceptual details that traditional metrics may miss.

Lower LPIPS scores indicate higher perceptual similarity between generated and real images, meaning they look more alike to human eyes. LPIPS is valuable for assessing image quality in tasks like super-resolution, where visual perception is crucial.

#### 2.3.4 Intensity differences in pixels

This metric, developed specifically for this study, is designed to accurately assess the variations in pixel intensities between the synthesized 3T images and the original 3T images. By focusing on pixel-level intensity differences, it provides a detailed comparison of how closely the generated images replicate the actual 3T scans. The goal is to capture discrepancies in intensity values, allowing for a thorough evaluation of image quality and ensuring that subtle differences between the synthesized and original images are quantified. This approach offers a comprehensive understanding of the accuracy and effectiveness of the synthesis process.


$$Q\,\left(i,j\right)=1\,i\,f\,\frac{\left|P_{\text{original}\,\left(i,j\right)}-P_{\text{synthesized}\,\left(i,j\right)}\right|}{P_{\text{original}\,\left(i,j\right)}}>\psi$$

$$I\,D\,P=\sum_{i}^{N}Q\,\left(i,j\right)/N;\psi =0\%,10\%,20\%,25\%,50\%,75\%$$

The IDP metric (Pham et al., 2017), (Chen et al., 2018) involves counting the number of pixels with varying levels of intensity differences between the synthetic and original 3T images, categorized into > 0%, > 10%, > 20%, > 50%, and > 75% differences. Then take the percentage of pixels with difference, determined by the five different thresholds, respectively, over the total pixel number.

### 2.4 Performance comparison

We have conducted pairwise comparison using paired test for each pair of SR models. The difference by each benchmark index and the associated 95% confidence interval was estimated using a mixed effect model.

We have identified two anchor points to determine the clinical significance: (1) The benchmark index between original 1.5 T and original 3 T. The benchmark index from SR models must be statistically significantly smaller than the baseline index. (2) The IDP between two repeated 1.5T scans in 9 subjects. The IDP between SR 3T and original 3T was expected to be higher than this baseline IDP, but closer the better.

## 3 Results

**FIGURE 6:**
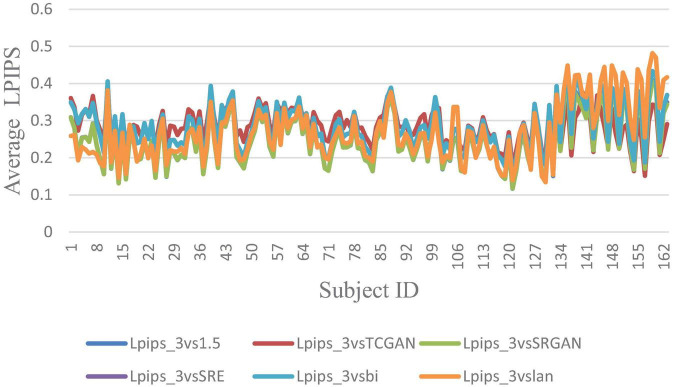
Average LPIPS across 163 Patient shows negligible differences between original 1.5T vs. 3T and synthetic 1.5T vs. 3T comparisons. Here, Lpips_3vsTCGAN represents the comparison between TCGAN-generated synthetic images and original 3T images. Similarly, Lpips_3vsSRGAN, Lpips_3vsSRE, Lpips_3vsBi, and Lpips_3vslan indicate comparisons between original 3T images and their respective methods. Lpips_3vs1.5 compares original 1.5T T1-weighted images with 3T images.

**FIGURE 7:**
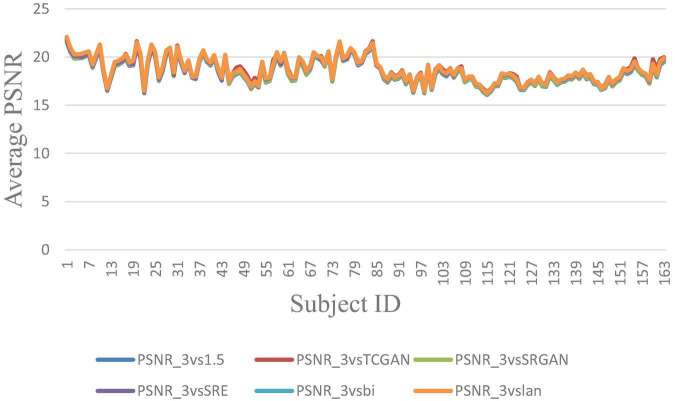
Average PSNR across 163 Patient shows negligible differences between original 1.5T vs. 3T and synthetic 1.5T vs. 3T comparisons. Here, PSNR_3vsTCGAN represents the comparison between TCGAN-generated synthetic images and original 3T images. Similarly, PSNR_3vsSRGAN, PSNR_3vsSRE, PSNR_3vsBi, and PSNR_3vslan indicate comparisons between original 3T images and their respective methods. PSNR_3vs1.5 compares original 1.5T T1-weighted images with 3T images.

**FIGURE 8:**
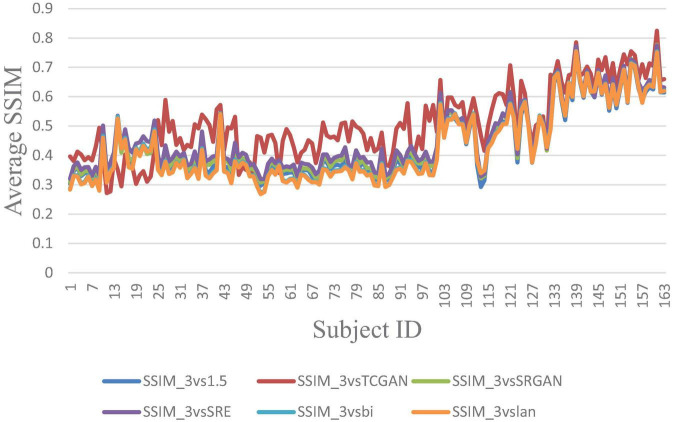
Average SSIM across 163 Patient shows negligible differences between original 1.5T vs. 3T and synthetic 1.5T vs. 3T comparisons. Here, SSIM _3vsTCGAN represents the comparison between TCGAN-generated synthetic images and original 3T images. Similarly, SSIM _3vsSRGAN, SSIM _3vsSRE, SSIM _3vsBi, and SSIM _3vslan indicate comparisons between original 3T images and their respective methods. SSIM _3vs1.5 compares original 1.5T T1-weighted images with 3T images.

The trends of SSIM, LPIPS and PSNR showed no significant improvement in Figures 6–8 but superiority of TCGAN model has been shown in 163 patients according to *p* < 0.001 (Supplementary Table 2).

However, a significant improvement in the Intensity Difference in Pixels (IDP) was observed with TCGAN (Transformer Enhanced GAN), SRGAN (Super-Resolution Generative Adversarial Network) and ESPCN (Efficient Sub-Pixel Convolutional Neural Network) compared to Bicubic, Lanczos, and the original comparison methods. TCGAN achieved the best performance among the SR methods evaluated (*p* < 0.001) (Supplementary Table 2).

**FIGURE 9:**
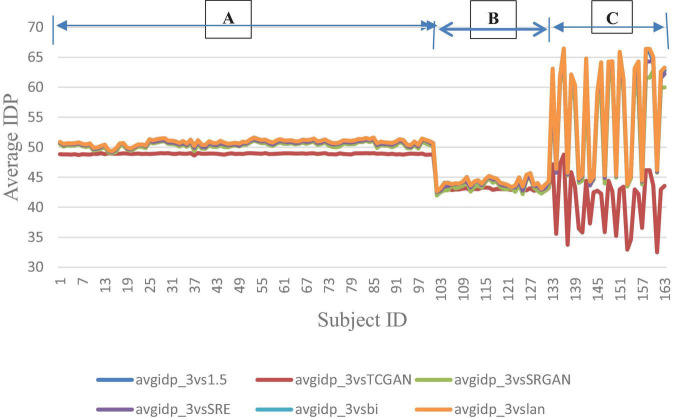
Average IDP across 163 patient shows negligible differences between original 1.5T vs. 3T and synthetic 1.5T vs. 3T comparisons. Here, avgidp_3vsTCGAN represents the comparison between TCGAN-generated synthetic images and original 3T images. Similarly, avgidp_3vsSRGAN, avgidp_3vsSRE, avgidp_3vsBi, and avgidp_3vslan indicate comparisons between original 3T images and their respective methods. avgidp_3vs1.5 compares original 1.5T T1-weighted images with 3T images A, B, and C indicate that 101, 31, and 31 subjects were collected using Siemens, GE, and Philips manufacturers, respectively.

**FIGURE 10:**
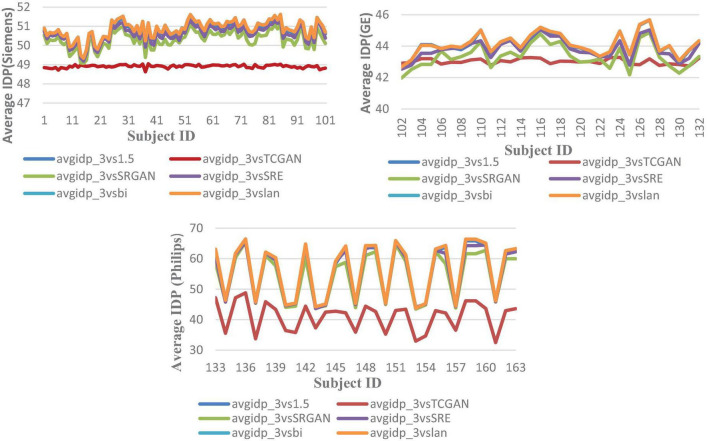
Average IDP across 163 patients, comparing original 1.5T vs. 3T images and synthetic 1.5T vs. 3T images. The IDP is shown in three manufacturers -specific charts. For all manufacturers, the IDP reveals significant differences when using the TCGAN method. Here, avgidp_3vsTCGAN represents the comparison between TCGAN-generated synthetic images and original 3T images. Similarly, avgidp_3vsSRGAN, avgidp_3vsSRE, avgidp_3vsBi, and avgidp_3vslan represent comparisons between original 3T images and their respective methods. avgidp_3vs1.5 compares original 1.5T T1-weighted images with 3T images.

Figure 9 presents the average IDP across 163 patients, combining data from all three vendors in a single chart. For further analysis, Figure 10 provides the IDP for each vendor separately. This analysis demonstrates that, irrespective of the vendor source, TCGAN consistently reduces the total number of pixels showing intensity-level differences when compared to the original images. To obtain these results, we calculated the pixel intensity difference for each image slice, averaged these values across all slices for each subject, and then averaged these results across all subjects. This approach highlights TCGAN’s effectiveness in generating synthetic images that closely align with the intensity distribution of original images, as shown in Figure 9. Pairwise comparisons using GEE model showed the statistical superiority of TCGAN compared to other methods (*p* < 0.001) (Supplementary Table 2).

**FIGURE 11:**
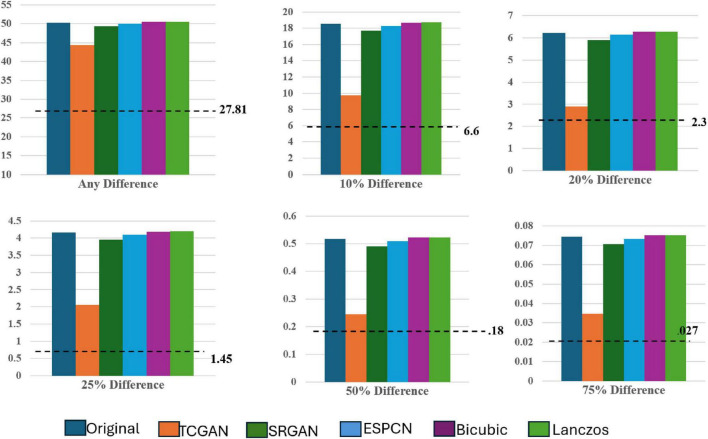
IDP across all SR models.

The dotted line denotes the Intensity Differences in Pixels (IDP) for a repeat scan, serving as a reference for variability due to measurement error (Figure 11). The figure contains six bar charts representing IDP values at different thresholds: 0, 10, 20, 25, 50, and 75%. These charts compare IDP across several super-resolution models, each evaluated against high field 3T MRI images to assess image quality consistency and detail preservation.

Focusing on the 0% IDP threshold in Figure 8, the bar chart indicates that, in the original comparison between 1.5T and 3T images, 32% of pixels exhibit non-zero differences, pointing to notable image disparities. TCGAN’s synthetic images, however, reduce this difference by 6%, illustrating its effectiveness in narrowing the visual and structural gaps between low-field (1.5T) and high-field (3T) MRIs. When considering the 27% repeat measurement error characteristic of 1.5T MRI scans, the net improvement of 6% is substantial, especially when considering the original 11.3% baseline difference between 1.5T and 3T scans. At other IDP thresholds, TCGAN’s performance continues to stand out. For instance, at the 75% threshold, where fewer details are required to match the higher field quality, TCGAN still manages to decrease the difference by 0.04%. This result highlights TCGAN’s consistent ability to enhance image quality even under less stringent comparison conditions, underscoring its robustness across different levels of image detail. An illustrative example using axial slices is presented below.

**FIGURE 12:**
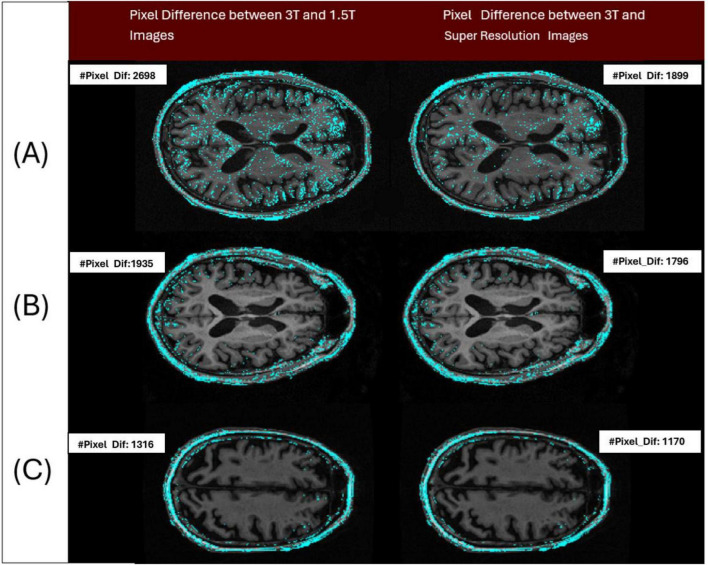
Three pairs of images acquired from the 3 patients imaged across 3 different vendors (Siemens/Philips/GE), have been analyzed to show the differences in intensity levels between 3T and 1.5T (original/super resolution) image pairs (cyan colored points). Pair A represents a sample image pair from Siemens Scanner. The left image of the pair shows the number of pixels with at least a 1 gray level difference in intensity between 3T and 1.5T (IDP = 2,698 pixels) and the right image of the pair shows the number of pixels with at least a 1 gray level difference in intensity between 3T and super resolution image (IDP = 1,899 pixels). Pair B and C represent the samples from Philips and GE Scanners, respectively. By design, a lower IDP represents a closer match between the image pairs. Here in all cases, the image pair with the super resolution 1.5T showed a reduction in IDP compared to the original pair.

A notable improvement (Figure 12) in IDP metrics was seen when comparing the original image pair to the image pair with super resolution 1.5T image. This improvement was observed on GE, Siemens, and Philips scanners. This improvement highlights the model’s capability to generate synthetic images with reduced pixel intensity discrepancies relative to the original 3T scans. The displayed slices serve as representative examples, demonstrating how TCGAN generated 1.5T images effectively minimizing distortions and structural inconsistencies compared to the original 1.5T images.

## 4 Discussion

Our research demonstrated a substantial improvement in synthesizing high-quality 3T MRI images from lower-resolution 1.5T MRI scans through advanced super-resolution (SR) techniques, especially using the Transformer-Enhanced Generative Adversarial Network (TCGAN). To harmonize image quality, we examined two primary approaches: interpolation and super-resolution (SR). Interpolation estimates pixel values by averaging nearby pixels, offering a simple and computationally light method suited to real-time applications where speed is prioritized over fine detail. Conversely, SR employs deep neural networks to generate high-resolution images from low-resolution inputs, creating a refined output with enhanced structural and textural detail. This approach is optimal for applications where high image quality is essential, as it can recover fine details and reduce artifacts common in low-resolution images. Our findings highlight that SR methods provide superior image quality and detail, making it preferable when precision is critical.

Among SR methods, as per the IDP analysis, TCGAN outperformed all its counterparts. Our findings demonstrate TCGAN’s superior ability to improve image fidelity, providing a more uniform and reliable representation across varying field strengths and scan conditions, and enhancing the utility of 1.5T scans in clinical settings. While we do not have a direct comparison, Turkowski (1990) generated synthetic CT images from PET images using TCGAN, demonstrating TCGAN’s superior accuracy compared to other GAN-based methods. They further validated its performance across three additional datasets, showing significant improvements in error minimization when comparing synthetic images to the originals. The improved performance of TCGAN may be attributed to its strength from utilizing a combination of a CNN and transformer architecture. Incorporating a transformer generator enhances the GAN’s image synthesis capacity. The transformer overcomes the limitations of CNN, which has a restricted local field of view, by attending to a wider region, thus identifying correlations between the target region and surrounding areas. Also, the patch-based Discriminator and combination of three loss functions help in capturing intricate details and nuances within the MRI images, thereby effectively reducing pixel differences and enhancing overall image quality. By generating synthetic images that closely mimic the characteristics of 3T image quality, TCGAN addresses the limitations of lower-resolution data, allowing for a more detailed visualization of anatomical structures and pathologies (Graves, 2022).

In our work, we also assessed the efficacy of various SR methods, utilizing full-reference image quality metrics such as Structural Similarity Index (SSIM) and Peak Signal-to-Noise Ratio (PSNR), along with the Learned Perceptual Image Patch Similarity (LPIPS) metric to understand perceptual similarity between synthesized images and high-quality 3T MRI scans. Interestingly, these traditional metrics showed no significant difference across the SR methods when compared to original 3T images. While SSIM and PSNR are widely used for assessing image quality, they often fall short in capturing subtle perceptual differences crucial in clinical contexts. SSIM, though effective at evaluating structural information, can miss fine variations in texture and detail—elements vital for accurately identifying nuanced diagnostic features. PSNR, similarly, lacks sensitivity to subtle distortions, especially in cases where low-level image noise significantly impacts diagnosis. As expected, our developed Intensity Difference in Pixels (IDP) metric, designed specifically to address the limitations of traditional metrics by focusing on subtle textural and structural distinctions critical for diagnostic applications showed differences between the deep learning versus interpolation methods of achieving super-resolution. Using IDP metric, we report that the average number of pixel intensity differences has been reduced significantly using TCGAN synthesized images. Also, for 10, 20, 25, 50, and 75% it was shown that TCGAN not only reduced the average number of pixels with intensity differences, but it also showed that the difference in value is much lower in comparison to other SR methods. In summary, TCGAN closely imitates the original 3T image quality compared to other SR methods namely, SRGAN, ESPCN, Bicubic and Lanczos.

Czobit and Samavi (2024) shows that CycleGan performed well to improve the quality of synthetic images, but this study does not utilize the same patient or the same scanner for 1.5T and 3T images when evaluating the performance of synthetic images. Additionally, it only considers 10 slices per patient, resulting in a total of 350 slices for 3T and 160 slices for 1.5T. In contrast, our study utilizes the same patient, same scanner 1.5T and 3T images for our evaluation. In addition, we have considered all 170 slices for 200 patients and calculated the average IDP for each patient, demonstrating the robustness of our approach. Furthermore, we performed rigorous quality control, removing cases with artifacts to ensure data integrity. Unlike this study, we also established a baseline by analyzing same-patient, same-day, same-scanner 1.5T scans to determine the acceptable IDP variation.

## 5 Conclusion and future work

Our research demonstrated a notable improvement in synthesizing high-quality 3T MRI images from lower-resolution 1.5T scans, particularly using the Transformer Enhanced Generative Adversarial Network (TCGAN). TCGAN enabled us to produce images closely resembling direct 3T MRI scans. Interestingly, interpolation methods performed comparable to SR methods except TCGAN for 1.5T-3T harmonization in our dataset. This observation may stem from the fact that the analyzed 1.5T-3T pair were acquired by the Alzheimer’s Disease Neuroimaging Initiative (ADNI) to facilitate direct comparisons between the two different magnetic field strengths. This unique dataset allows us to assess the variations in image quality, detail, and diagnostic utility that arise from the differing field strengths. Paired-imaging studies such as ours using real world data are warranted.

Some of the limitations of our study are:

1. The effectiveness of TCGAN is dependent on the quality of the input data, as significant noise or artifacts in the lower-resolution 1.5T scans may be propagated into the synthesized 3T images, limiting the technique’s overall performance. We also performed quality control on all MRI images in our dataset, removing artifacts such as motion, ringing etc. Additionally, we applied bias correction to address inhomogeneity and ensured that the dataset does not contain any artifacts related issues.
2. We only considered data from the same manufacturers, as cross- manufacturer evaluation yielded suboptimal results for different imaging protocols. However, our lab is also working on manufacturer-based data harmonization.
3. In a sub-analysis, we observed a decline in IDP performance metrics when generating synthetic images across vendors, highlighting the model’s sensitivity to vendor-specific acquisition characteristics. To further assess potential hallucinated artifacts and improve synthetic image generation, we plan to conduct texture-based analyses in future work, assessing the robustness and reliability of synthetic image generation in minimizing these artifacts.

While the super-resolution images generated through TCGAN demonstrate promising improvements, rigorous validation is required. Our future goal is to validate the effectiveness of super-resolution images generated by TCGAN for use in radiomics and other downstream tasks. Radiomics plays a crucial role in clinical applications like risk stratification and treatment response prediction by extracting quantitative features from medical images. To ensure the practical utility of these synthetic images, we need to confirm that they accurately preserve the quantitative features vital for radiomic analyses. This validation process will focus on evaluating the performance of TCGAN-enhanced images in key clinical areas, including diagnosis, treatment planning, and disease monitoring.

In conclusion, the main contribution of this paper is that SR techniques can help in harmonizing the image quality differences between 1.5T and 3T scans. In this effort, TCGAN shows superior performance compared to other SR techniques. While further validation using large sample size and real-world data is warranted, our results support the idea that the implementation of SR based harmonization in MRI imaging offers substantial benefits for quantitative imaging techniques such as radiomics by improving image quality consistency. This improvement can in turn lead to enhancing feature consistency and increasing the robustness of predictive models. These improvements are vital for accurate disease diagnosis and treatment planning, ultimately leading to better clinical outcomes.

## Data Availability

Publicly available datasets were analyzed in this study. This data can be found here: https://adni.loni.usc.edu/data-samples/adni-data/.

## Footnotes

1
https://healthimaging.com/topics/medical-imaging/diagnostic-screening/why-15tmri-leading-pack

2
https://healthimaging.com/topics/medical-imaging/diagnostic-imaging/15t-mrifriend-physician-and-patient-alike
